# Heparanase-induced shedding of syndecan-1/CD138 in myeloma and endothelial cells activates VEGFR2 and an invasive phenotype: prevention by novel synstatins

**DOI:** 10.1038/oncsis.2016.5

**Published:** 2016-02-29

**Authors:** O Jung, V Trapp-Stamborski, A Purushothaman, H Jin, H Wang, R D Sanderson, A C Rapraeger

**Affiliations:** 1 Department of Human Oncology, Wisconsin Institutes for Medical Research, Carbone Cancer Center, University of Wisconsin-Madison, Madison, WI, USA; 2 Graduate Program in Molecular and Cellular Pharmacology, School of Medicine and Public Health, University of Wisconsin-Madison, Madison, WI, USA; 3 Department of Pathology, Wallace Tumor Institute, UAB Comprehensive Cancer Center, University of Alabama at Birmingham, Birmingham, AL, USA

## Abstract

Multiple myeloma arises when malignant plasma cells invade and form multiple tumors in the bone marrow. High levels of heparanase (HPSE) correlate with poor prognosis in myeloma patients. A likely target of the enzyme is the heparan sulfate (HS) proteoglycan syndecan-1 (Sdc1, CD138), which is highly expressed on myeloma cells and contributes to poor prognosis in this disease. We find that HPSE promotes an invasive phenotype mediated by the very late antigen-4 (VLA-4, or α4β1 integrin) in myeloma cells plated on either fibronectin (FN) or vascular endothelial cell adhesion molecule-1 (VCAM-1), ligands that are prevalent in the bone marrow. The phenotype depends on vascular endothelial cell growth factor receptor-2 (VEGFR2), which is aberrantly expressed in myeloma, and is characterized by a highly protrusive lamellipodium and cell invasion. HPSE-mediated trimming of the HS on Sdc1 and subsequent matrix metalloproteinase-9-mediated shedding of the syndecan exposes a juxtamembrane site in Sdc1 that binds VEGFR2 and VLA-4, thereby coupling VEGFR2 to the integrin. Shed Sdc1 can be mimicked by recombinant Sdc1 ectodomain or by a peptide based on its binding motif, which causes VLA-4 to re-orient from the lagging edge (uropod) to the leading edge of migrating cells, couple with and activate VEGFR2. Peptides (called 'synstatins') containing only the VLA-4 or VEGFR2 binding sites competitively inhibit invasion, as they block coupling of the receptors. This mechanism is also utilized by vascular endothelial cells, in which it is also activated by HPSE, during endothelial cell tube formation. Collectively, our findings reveal for the first time the mechanism through which HPSE modulates Sdc1 function to promote both tumor cell invasion and angiogenesis, thereby driving multiple myeloma progression. The inhibitory synstatins, or inhibitors of HPSE enzyme activity, are likely to show promise as therapeutics against myeloma extravasation and spread.

## Introduction

Multiple myeloma, a disease in which malignant plasma cells form disruptive bone tumors, is the second most prevalent hematologic malignancy in the United States.^1, 2^ The emergence of new therapies (for example, bortezomib and thalidomide) has greatly improved survival rates in myeloma patients.^2^ However, these therapies slow rather than cure the disease and patients ultimately develop resistance and become refractory. Thus, the discovery of additional mechanisms involved in disease progression that can be targeted by new therapies remains a high priority.

Heparanase (HPSE), an endo-β-d-glucuronidase that degrades heparan sulfate (HS) glycosaminoglycan chains, is a tumor promoter in multiple myeloma, as well as in many other cancers.^3, 4, 5, 6^ It is thought that the HS fragments released by HPSE bind and promote the activity of heparin-binding growth factors and alter the expression of genes that affect the proliferation, invasion and survival of tumor cells and other cells in the tumor microenvironment.^5, 6, 7^ A major target of HPSE in multiple myeloma is syndecan-1 (Sdc1, CD138), one of a family of cell surface HS proteoglycans found on most cells. Sdc1 is highly expressed on malignant plasma cells and has a causal role in multiple myeloma.^8, 9, 10, 11, 12, 13, 14^

Pruning of its HS chains by HPSE causes matrix metalloproteinase-9 (MMP-9)-mediated shedding of Sdc1 ectodomain into the tumor microenvironment where the proteoglycan enhances angiogenesis and is likely to have roles in myeloma cell adhesion, proliferation, metastasis and survival.^11, 12, 15, 16, 17, 18, 19, 20^ Indeed, high levels of shed Sdc1 in patient serum correlate with poor prognosis.^21, 22, 23^ Although Sdc1 is shed, the steady-state level of cell surface Sdc1 remains unchanged because of a HPSE-induced increase in receptor expression.^15, 16, 19^ Thus, Sdc1 exists in at least two functional states in myeloma—a cell surface receptor and a bioactive agent in the extracellular milieu. But whether it is the cell surface or the shed form of Sdc1 that mediates the potent effect of HPSE on myeloma progression is not clear. As a cell surface receptor, Sdc1 has been shown to organize integrin and growth factor receptor signaling.^24, 25^ The best-characterized example involves the insulin-like growth factor-1 receptor (IGF-1R) and the αvβ3- or αvβ5 integrin in carcinoma and activated endothelial cells.^26, 27^ These receptors are captured by an active site in the syndecan extracellular domain (amino acids 93–120 in human), which promotes activation of IGF-1R and inside-out signaling that activates the integrins.^26, 28^ An inhibitory peptide that mimics the capture site in Sdc1, called a synstatin (synstatin 93–120 or SSTN IGF-1R (SSTN^IGF1R^)), disrupts the assembly of the receptor complex, blocks tumor growth and tumor-induced angiogenesis, and is a candidate for therapeutic intervention in human disease.^24^

Given the emerging role of Sdc1 as an organizer of matrix- and growth factor-dependent signaling, we speculated that the tumor-promoting activity of HPSE may trace to its activation of such a mechanism during myeloma cell adhesion and invasion. We report here that myeloma cells bind fibronectin (FN) and vascular endothelial cell adhesion molecule-1 (VCAM-1), abundant ligands for very late antigen-4 (VLA-4) in the bone marrow with roles in myeloma growth, survival and extravasation,^29, 30^ and that cells expressing elevated levels of HPSE adopt an invasive phenotype on these ligands because of HPSE-mediated shedding of Sdc1, which couples vascular endothelial cell growth factor receptor-2 (VEGFR2) to VLA-4, activating its kinase activity. Importantly, the mechanism is also present on vascular endothelial cells. Coupling mediated by Sdc1 traces to an active site in the Sdc1 core protein comprises amino acids 210–236. A peptide based on this site (S1ED^210–236^) also couples VEGFR2 to VLA-4 and duplicates the activity of shed Sdc1. However, shorter peptides, containing the binding motif for only VEGFR2 or VLA-4, act as inhibitory 'synstatins' by preventing receptor coupling and inhibiting myeloma cell invasion and endothelial cell tube formation *in vitro*.

## Results

### Adhesion and spreading of CAG cells on FN or VCAM-1 is enhanced by HPSE

**Figure 1:**
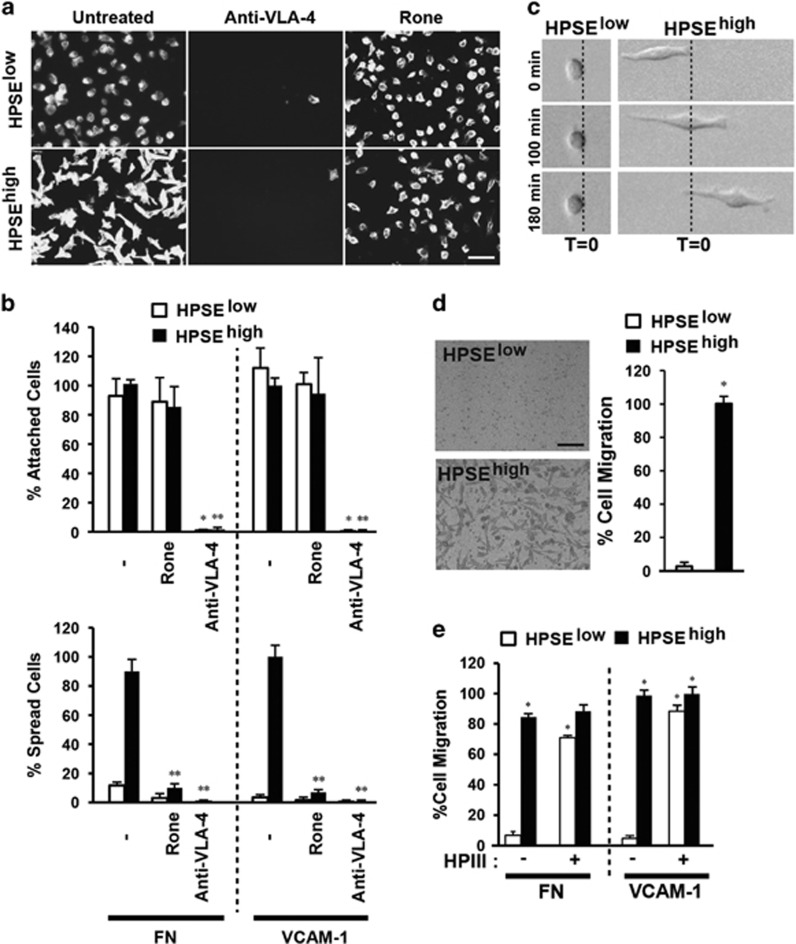
Polarized migration of CAG cells on FN or VCAM-1 is enhanced by HPSE. (**a**) HPSE^low^ or HPSE^high^ cells were plated on VCAM-1 with or without treatment with 500 μg/ml Roneparstat (Rone) or 10 μg/ml VLA-4-blocking antibody (P1H4) and stained with fluorescent phalloidin to visualize the cells (bar=50 μm). (**b**) Quantification of HPSE^low^ and HPSE^high^ cell attachment and spreading on FN or VCAM-1 in the presence of inhibitors. *P<0.05 against untreated HPSE^low^ cells. **P<0.05 against untreated HPSE^high^ cells. (**c**) Pictures of representative HPSE^low^ or HPSE^high^ cells at *t*=0, 100 and 180 monitored by time-lapse microscopy on VCAM-1. (**d**) Representative pictures after 12-h migration of HPSE^low^ cells or HPSE^high^ cells through FN-coated filters. Cells on the bottom of the filter in five random images for each experiment are quantified as a percent of HPSE^high^ cell migration (bar=100 μm). *P<0.05 against HPSE^low^ cells. (**e**) Quantification of HPSE^low^ and HPSE^high^ cell migration toward FN or VCAM-1 in the presence of heparitinase III after 16-h migration. Cells on the bottom of the filter in five random images for each experiment are quantified as a percent of untreated HPSE^high^ cell migration toward VCAM-1. *P<0.05 against untreated HPSE^low^ cells. Error bars represent s.e.

Prior work using CAG myeloma cells as a model for tumor formation *in vivo* has revealed a strong correlation between tumor growth and expression of HPSE and Sdc1.^4, 6, 8, 11, 15^ To identify a potential link between these two effectors, we examined the effects of high HPSE expression on CAG adhesion and motility on FN or VCAM-1, two ligands enriched in the bone marrow that are recognized by VLA-4 integrin expressed by these cells. CAG cells expressing low (HPSE^low^) or high (HPSE^high^) amounts of HPSE attach equally via VLA-4 to FN or VCAM-1 (Figures 1a and b). However, cells expressing high levels of HPSE form a highly polarized morphology within 2.5 h (Figures 1a and b). Although HPSE is known to affect cell adhesion, including activation of β1 integrins, via both enzymatic and nonenzymatic means,^7, 31, 32, 33, 34^ myeloma spreading requires enzymatic activity because it is blocked by pre-treatment with the HPSE inhibitor Roneparstat^35, 36, 37^ (Figures 1a and b), and cannot be induced by enzymatically inactive HPSE^M225^ (ref. 15) overexpressed in the cells (Supplementary Figure S1). Prolonged expression, which can alter gene expression,^17, 38^ is not required because addition of exogenous HPSE during the 2.5-h assay is sufficient to promote spreading (Supplementary Figure S2), as is treatment with heparinase III (HPIII), a structurally unrelated bacterial enzyme that also degrades the HS^39^ (Supplementary Figure S3).

The polarized morphology displayed by the HPSE^high^ cells is typical of motile cells.^40, 41^ Indeed, HPSE^high^ cells, but not HPSE^low^, display directional migration because of formation of a highly active lamellipodium at their leading edge (Figure 1c). Furthermore, large numbers of polarized HPSE^high^ cells invade through a FN-coated filter compared with few, if any, HPSE^low^ cells (Figure 1d) or cells expressing HPSE^M225^ (Supplementary Figure S1). Exogenous HPIII also stimulates invasion (Figure 1e), again demonstrating the link between invasion and HS trimming.

### Shed Sdc1 mediates the HPSE-enhanced effect in CAG cells

**Figure 2:**
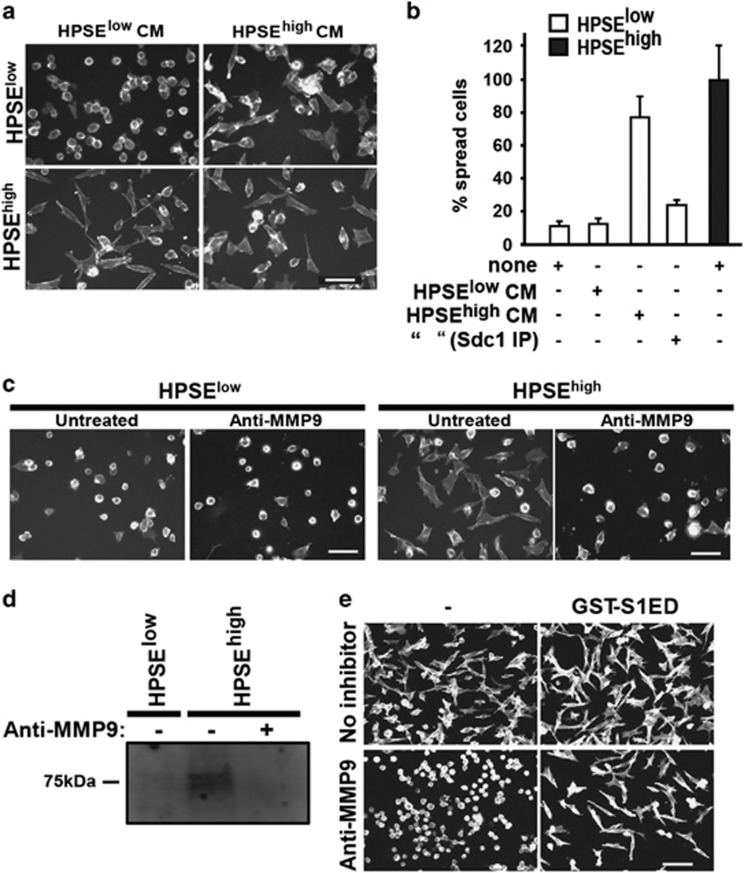
MMP-9-mediated shedding of Sdc1 triggers the invasive phenotype. (**a**) The polarized spreading of HPSE^low^ cells on FN was tested in the presence or absence of medium conditioned for 24 h by HPSE^low^ or HPSE^high^ cells. HPSE^high^ cell spreading with both treatments is shown as a control. Bar=50 μM. (**b**) Quantification of spreading of HPSE low cells in the presence or absence of medium conditioned for 24 h by HPSE^low^ or HPSE^high^ cells, or conditioned medium from HPSE^high^ cells in which Sdc1 was immunodepleted. Spreading is expressed as a percentage of spreading by untreated HPSE^high^ cells. (**c**) HPSE^low^ and HPSE^high^ cells are plated on VCAM-1 in the absence or presence of 10 μg/ml MMP-9 inhibitory antibody (bar=50 μm). (**d**) HPSE^low^ and HPSE^high^ cells were grown at equal densities in serum-free media for 48 h in the absence or presence of MMP-9 blocking antibody, followed by immunoblotting for shed Sdc1 in the conditioned media. (**e**) HPSE^high^ cells were plated on FN in the absence or presence of MMP-9 blocking antibody with or without treatment with 4 μM GST-S1ED (bar=100 μm).

Trimming of the HS on Sdc1 is known to induce MMP-9-mediated shedding of the proteoglycan.^17, 19^ Testing whether shed Sdc1 triggers invasion, we find that medium conditioned by HPSE^high^ cells, which contains approximately 500 ng/ml Sdc1,^18^ induces the polarized phenotype in HPSE^low^ cells and is abolished if Sdc1 is immunodepleted (Figures 2a and b). Furthermore, an MMP-9 blocking antibody effectively blocks the polarized spreading of HPSE^high^ cells (Figure 2c), which correlates with reduced Sdc1 in the conditioned medium (Figure 2d). Next, we reasoned that if the mechanism relied on an active site in the shed Sdc1 protein, recombinant Sdc1 ectodomain (GST-S1ED) should promote the spreading even if Sdc1 shedding is blocked. Indeed, the polarized phenotype is rescued when GST-S1ED is added to HPSE^high^ cells in which MMP-9 activity and shedding is blocked (Figure 2e). Note that the concentration of recombinant S1ED is substantially higher than shed Sdc1 in the medium. This is partly due to the probability that a portion of S1ED purified from bacteria is not native, and partly because posttranslational modification of the native Sdc1, such as the remaining HS chains, may assist in stabilizing the binding. A similar inhibition is observed using MMP-9 inhibitor during spreading of HPSE^low^ cells induced by HPIII, which is also rescued with GST-S1ED (Supplementary Figure S4).

### Sdc1 extracellular domain contains a juxtamembrane active site

**Figure 3:**
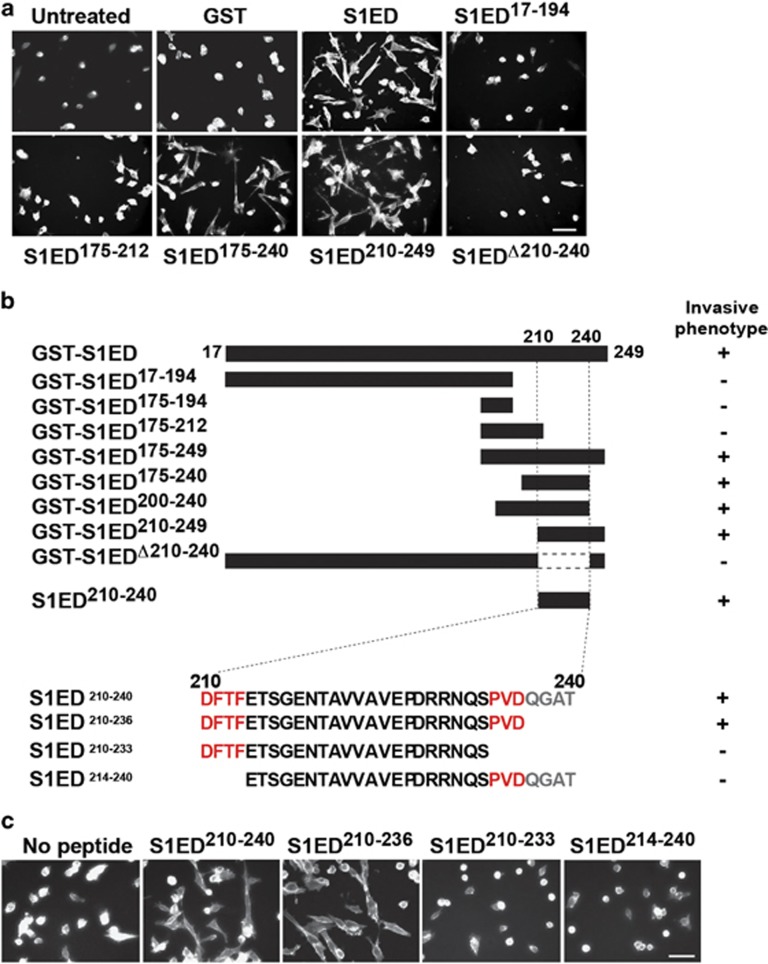
A juxtamembrane site in the Sdc1 ectodomain mimics the effects of HPSE. (**a**) HPSE^low^ cells were plated on VCAM-1 and treated with 4 μM GST-S1ED constructs shown (bar=50 μm). (**b**) Schematic presentation of GST-tagged S1ED constructs and S1ED peptides used and a summary of their ability to induce spreading indicative of the invasive phenotype. (**c**) HPSE^low^ cells were plated on VCAM-1 in the absence or presence of 0.3 μM S1ED^210-240^, S1ED^210-236^, S1ED^210-233^ or S1ED^214-240^ peptide (bar=50 μm).

To identify the putative active site in Sdc1, a library of GST-S1ED fusion proteins was tested for their ability to induce the polarized phenotype (Figure 3). Fusion proteins retaining a juxtamembrane site demarcated by amino acids 210–240 retains induction activity, whereas those lacking this region do not (Figures 3a and b). Furthermore, a synthetic peptide comprising amino acids 210–240 (S1ED^210-240^) fully promotes the spreading activity (Figures 3b and c); again note that synthetic peptides function at lower concentrations, likely reflecting denaturation of GST-S1ED purified from bacteria. Recent work has identified this region as a capture site for HER2 and α3β1 integrin in epithelial cells, in which S1ED^210-240^ blocks HER2-stimulated motility;^25^ HER2 capture depends on a highly conserved DFTF motif at the N-terminus of the peptide, whereas α3β1 integrin depends on QGAT at the C-terminus.^25^ Screening peptides encompassing this region in the myeloma cell spreading assay, we find that truncation of the QGAT motif (S1ED^210-236^) is without effect, whereas further truncation of a conserved PVD motif (S1ED^210-233^) disrupts cell spreading, as does removal of the N-terminal DFTF motif (S1ED^214-240^) (Figures 3b and c).

### Sdc1 causes cell adhesion-dependent activation of VEGFR2

**Figure 4:**
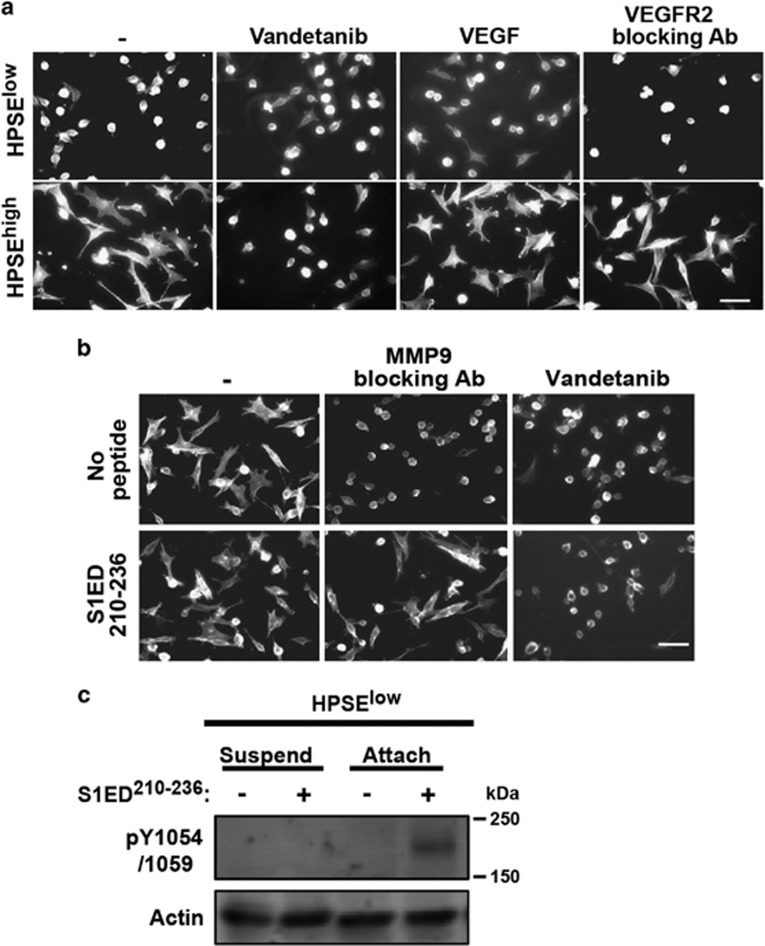
HPSE causes activation of VEGFR2 when VLA-4 engages ligand. (**a**) HPSE^low^ and HPSE^high^ cells treated with or without 1 μM Vandetanib, 20 ng/ml VEGF or 20 μg/ml VEGFR2 blocking antibody are plated on VCAM-1 to observe cell spreading (bar=50 μm). (**b**) HPSE^high^ cells plated on FN were treated with MMP-9 blocking antibody or 1 μM Vandetanib in the absence or presence of 0.3 μM S1ED^210-236^ (bar=50 μm). (**c**) HPSE^low^ cells were kept in suspension or were seeded on FN in the absence or presence of 0.3 μM S1ED^210-236^ for 2.5 h. Cell lysates were immunoblotted with antibodies against pY1054/1059 in VEGFR2 as a marker of VEGFR2 activation. Actin is shown as a loading control.

Next, we questioned how the Sdc1 active site promotes the invasive phenotype. Based on our prior work,^24, 25, 26, 42^ we hypothesized that it organizes integrins and receptor tyrosine kinases into a signaling complex. To test this, we treated HPSE^high^ cells with kinase inhibitors in an attempt to block the mechanism, discovering that the VEGFR2 inhibitors vandetanib or VEGFR2 kinase inhibitor II (data not shown) inhibit HPSE^high^ cell spreading (Figure 4a). Surprisingly, however, addition of VEGF fails to induce spreading, and VEGFR2 blocking antibody fails to disrupt it (Figure 4a), identifying this as a VEGF-independent mechanism.

We next questioned whether active VEGFR2 lies upstream of Sdc1 (for example, responsible for shedding) or downstream (a potential target of Sdc1). As observed earlier with GST-S1ED, S1ED^210-236^ rescues spreading of HPSE^high^ cells in the presence of MMP-9 inhibitor (Figure 4b). However, it fails to rescue spreading blocked by vandetanib (Figure 4b), suggesting that VEGFR2 is activated downstream of shed Sdc1. Next, VEGFR2 activation was assessed by monitoring phosphorylation of Y1054/1059 in its kinase activation loop^43^ in the presence of S1ED^210-236^. VEGFR2 phosphorylation is not induced when suspended HPSE^low^ cells are treated with the peptide (Figure 4c, left). However, VEGFR2 is activated by the peptide when cells are plated on FN (Figure 4c, right), to which they adhere via VLA-4 (*cf.*, Figure 1b).

### Shed Sdc1 causes capture of VEGFR2 by VLA-4

**Figure 5:**
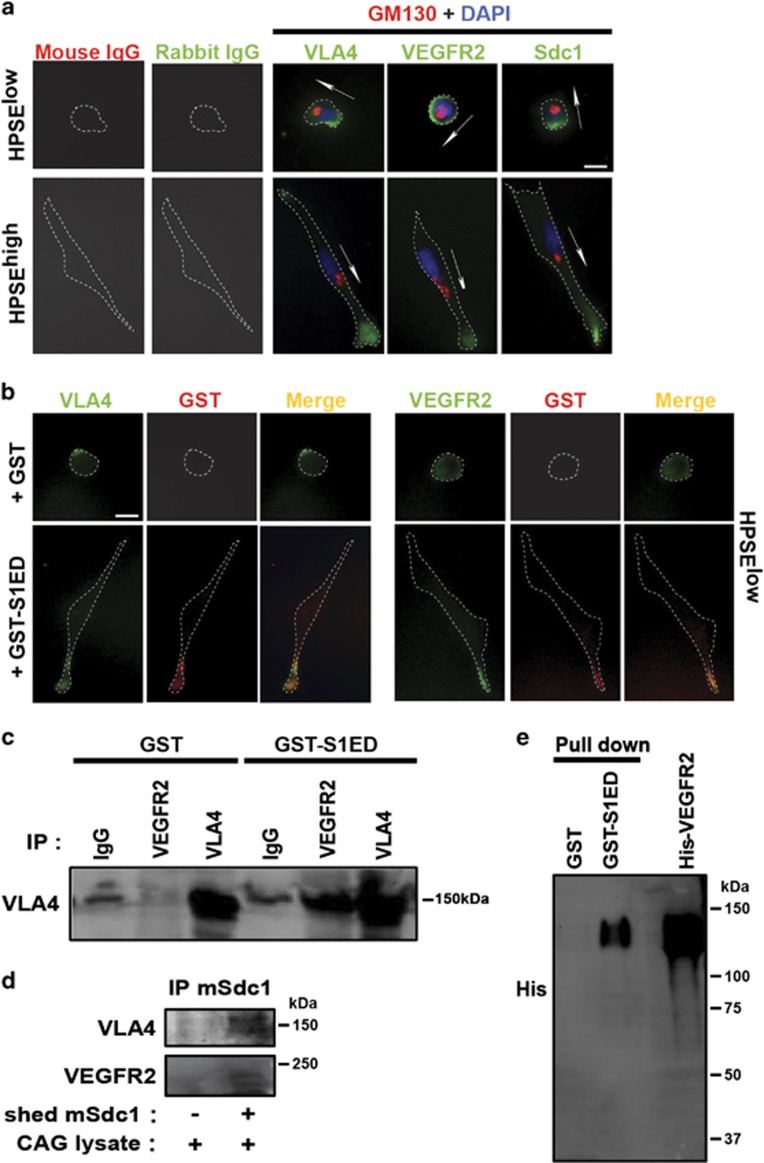
Shed Sdc1 causes capture of VEGFR2 by VLA-4. (**a**) HPSE^low^ cells and HPSE^high^ cells plated on FN for 2.5 h were stained with antibodies specific for VLA-4, VEGFR2, or Sdc1 (green) with GM130 (red), and DAPI (blue). Arrows denote anterior polarity based on position of the Golgi relative to the nucleus (bar =10 μm). (**b**) HPSE^low^ cells plated on FN in the presence of 4 μM GST or GST-S1ED for 2.5 h were double stained to detect bound GST (left) or GST-S1ED (right) compared with VLA-4 or VEGFR2 (bar=10 μm). (**c**) HPSE^low^ cells were plated on FN for 2.5 h in the absence or presence of GST-S1ED, then whole-cell lysates were subjected to immunoprecipitation with anti-VEGFR2, anti-VLA-4, or nonspecific IgG. Precipitated VLA-4 was detected by immunoblotting with anti-VLA-4 antibody. (**d**) Mouse Sdc1 from conditioned media of HPSE^high^ cells transfected with mouse Sdc1 was immobilized to mAb281.2-coated beads, incubated with CAG cell lysates and precipitated. Blots were probed for co-precipitation of VLA-4 and VEGFR2. (**e**) Recombinant His-tagged VEGFR2 protein was precipitated using beads bearing GST or GST-S1ED and detected on blots with anti-His antibody.

These findings suggest that shed Sdc1 couples VEGFR2 to VLA-4, which, when engaged by ligand, clusters and activates VEGFR2. To test this, we assessed the localization of VLA-4, Sdc1 and VEGFR2 when CAG cells engage FN. Sdc1 has been shown to localize to the uropod of myeloma cells.^44, 45^ Similarly here, VLA-4 and Sdc1 are polarized to the lagging edge (uropod) in HPSE^low^ cells, defined by their centripetal localization when compared with the Golgi (Figure 5a). In contrast, VEGFR2 is observed on the entire cell surface. However, VLA-4, VEGFR2 and Sdc1 (likely the shed extracellular domain) are oriented to the leading edge of HPSE^high^ cells (Figure 5a). Furthermore, in adherent HPSE^low^ cells treated with GST-S1ED to induce the polarized cell morphology, GST-S1ED also colocalizes with VEGFR2 and VLA-4 (Figure 5b). It appears that the receptors physically assemble with the Sdc1 extracellular domain, because VEGFR2 immunoprecipitated from HPSE^low^ cell lysates captures VLA-4 when GST-S1ED is provided (Figure 5c), and shed mouse Sdc1 ectodomain, expressed and isolated from the conditioned medium of HPSE^high^ cells, also captures both VLA-4 and VEGFR2 from CAG cell lysates (Figure 5d). Furthermore, the binding between Sdc1 and VEGFR2 is direct, as recombinant GST-S1ED captures purified, recombinant VEGFR2 extracellular domain (Figure 5e). In sum, these data suggest that shed Sdc1 activates VEGFR2 and ensuing VLA-4-dependent protrusive activity by colocalizing the kinase and the integrin at the leading edge of the cell.

### Sdc1-derived peptides function as synstatins to block the invasive phenotype

**Figure 6:**
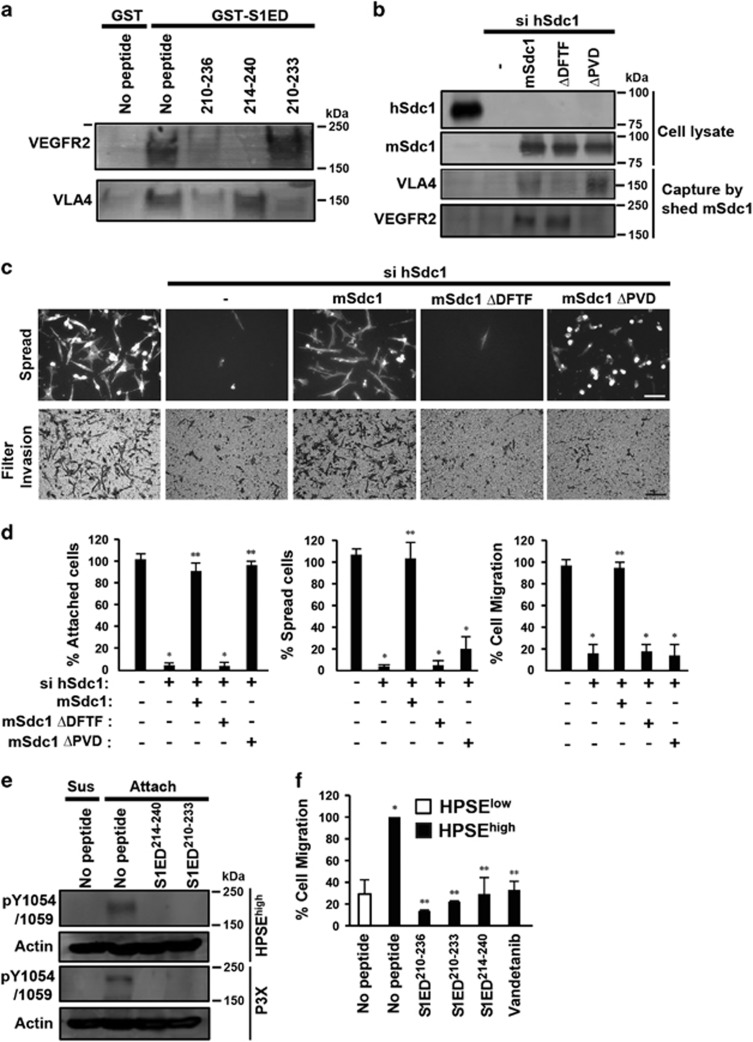
SSTN peptides specific for VEGFR2 or VLA-4 compete with shed Sdc1 and inhibit the HPSE-induced invasive phenotype. (**a**) HPSE^low^ cell lysates were incubated overnight with glutathione beads coated with GST or GST-S1ED in the absence or presence of 30 μM S1ED^210-236^, S1ED^214-240^ or S1ED^210-233^. Capture of VLA-4 or VEGFR2 was detected by immunoblotting. (**b**–**d**) HPSE^high^ cells were untreated, transfected with hSdc1 small interfering RNA (siRNA) alone or transfected with with hSdc1 siRNA together with cDNA for mSdc1, mSdc1^ΔDFTF^ or mSdc1^ΔPVD^ for 72 h, before performing immunoprecipitation, cell spreading or migration assays. (**b**) Top: blots were probed for hSdc1, mSdc1 or mSdc1 mutants following immunoprecipitation from whole-cell lysates. Bottom: Sdc1 captured from the conditioned medium of each cell treatment by mouse-Sdc1-specific mAb281-2-coated beads was incubated with CAG cell lysates, and probed on blots for co-precipitation of VLA-4 or VEGFR2. (**c**) Top: pictures of cell attachment and spreading following plating on FN for 2.5 h (bar=50 μm). Bottom: pictures after 12-h migration through FN-coated filters (bar=100 μm). (**d**) Quantification of cell attachment or spreading on FN and cell migration toward FN. *P<0.05 against untreated HPSE^high^ cells. **P<0.05 against HPSE^high^ cells transfected with hSdc1 siRNA. (**e**) HPSE^high^ or P3-X63-Ag8 cells were suspended or plated on FN in the absence or presence of 30 μM of S1ED^214-240^ or S1ED^210-233^. Cell lysates were immunoblotted with antibodies against pY1054/1059 in VEGFR2 as a marker of VEGFR2 activation. Actin is shown as a loading control. (**f**) Sixteen-hour transfilter migration assays toward FN were performed for HPSE^low^ or HPSE^high^ cells in the presence of 30 μM of S1ED^210-236^, S1ED^210-233^, S1ED^214-240^ or Vandetanib. Cells on the bottom of the filters were stained, imaged, quantified from five random images and expressed as percent migration compared with untreated HPSE^high^ cells. *P<0.05 against untreated HPSE^low^ cells. **P<0.05 against HPSE^high^ cells. Error bars represent s.e.

Next, we questioned which motif in S1ED^210-236^ is responsible for capture of VEGFR2 and VLA-4 by using truncated peptides as competitors in a pull-down assay. Whereas S1ED^210-236^ competes for the capture of both receptors from cell lysates by GST-S1ED, S1ED^214-240^ blocks only VEGFR2 capture and S1ED^210-233^ blocks VLA-4, identifying the DFTF and PVD motifs (*cf.* Figure 3) as required elements for VLA-4 and VEGFR2 capture, respectively (Figure 6a). To confirm this, we mutated these sites in mouse Sdc1, which was transiently expressed in HPSE^high^ cells. As expected, mSdc1^ΔDFTF^ and mSdc1^ΔPVD^ isolated from conditioned medium using mouse-specific mAb281.2 display reduced ability to capture VLA-4 and VEGFR2, respectively (Figure 6b). To test their effect on the invasive phenotype, the polarized spreading or invasion through FN-coated filters was analyzed after silencing the expression of endogenous hSdc1. Silencing hSdc1 expression blocked cell invasion, and, surprisingly, cell attachment as well, and both activities were rescued by mSdc1 (Figures 6c and d). mSdc1^ΔDFTF^, however, failed to rescue attachment, whereas mSdc1^ΔPVD^ rescued attachment, but failed to rescue polarized spreading or invasion (Figures 6c and d).

As this mechanism likely has a role in HPSE-enhanced myeloma disease progression, we determined using a range of peptide concentrations whether the truncated peptides that bind one, but not both, receptors can act as inhibitory 'synstatins' (SSTNs). S1ED^210-236^ induces spreading of HPSE^low^ cells at 0.3–3 μM concentrations, but loses this activity at 10–30 μM; this is mirrored by its blockade of HPSE^high^ cell spreading at 10–30 μM, suggesting that at high concentrations the peptide competes for, rather than couples, individual VEGFR2 and VLA-4 receptors (Supplementary Figure S5). Displacing Sdc1 from VLA-4 using S1ED^210-233^, which contains the DFTF motif that binds VLA-4, disrupts adhesion of the HPSE^low^ and HPSE^high^ cells with an IC_50_ of 10 μM without stimulating spreading (Supplementary Figure S5), qualifying it as a SSTN (SSTN^210-233^). S1ED^214-240^, which displaces VEGFR2 from Sdc1, has no affect on adhesion, but inhibits the polarized spreading of HPSE^high^ cells (Supplementary Figure S5), qualifying it as a SSTN as well (SSTN^214-240^). Note that the peptides are not cytotoxic because a 24-h preincubation in peptide does not prevent subsequent HPSE^high^ cell attachment and spreading (data not shown). Applying these inhibitory peptides to HPSE^high^ cells, we find that both block the activation of VEGFR2 mediated by VLA-4 engaged to FN (Figure 6e) and, when used at 30 μM concentrations, disrupt transfilter invasion of HPSE^high^ cells (Figure 6f). To extend these findings, we find that P3-X63-Ag8 myeloma cells also (i) display Sdc1-coupled VEGFR2 and VLA-4 (Supplementary Figure S6), (ii) activate VEGFR2 when attached to FN, which is blocked by the SSTN peptides (Figure 6e), (iii) bind and spread on FN, but fail to attach in the presence of SSTN^210-233^ or to spread in the presence of SSTN^214-240^ (Supplementary Figure S6) and (iv) fail to migrate through FN-coated filters in the presence of these peptides, or in the presence of VLA-4, VEGFR2 or HPSE inhibitors (Supplementary Figure S6).

### Sdc1 couples VEGFR2 to VLA-4 during endothelial cell tube formation *in vitro*

**Figure 7:**
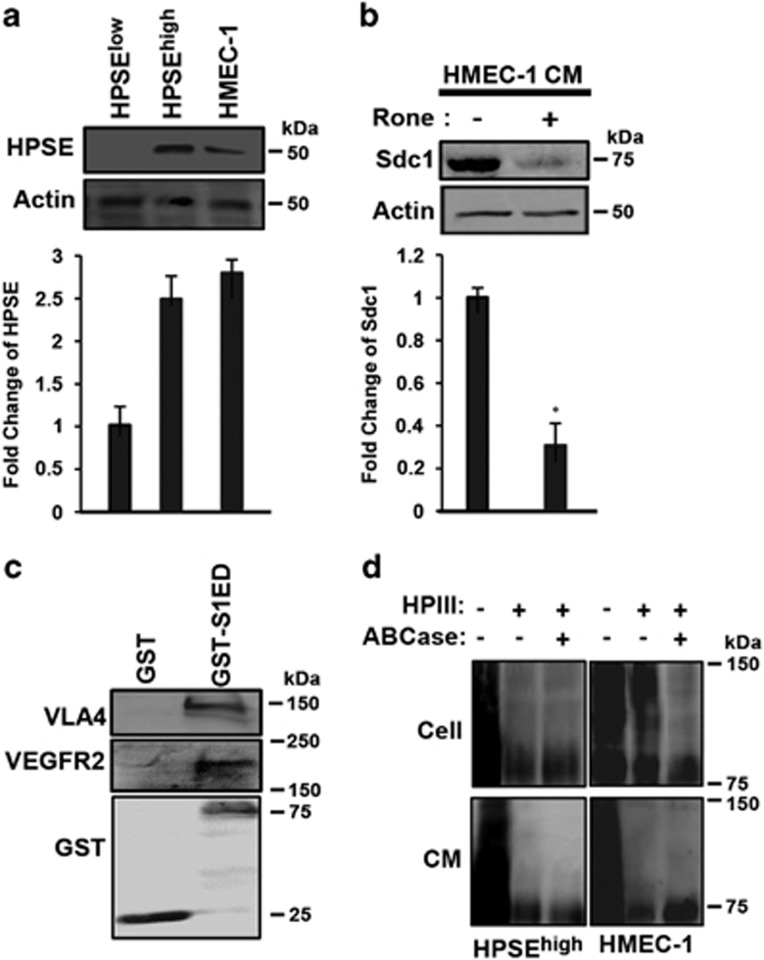
The endothelial cells express HPSE and shed CS-free Sdc1. (**a**) Lysates from HPSE^low^, HPSE^high^ and HMEC-1 cells are probed by immunoblotting for expression of HPSE (top) and quantified (bottom); (**b**) medium conditioned (CM) by HMEC-1 cells for 24 h in the absence or presence of 125 μg/ml Roneparstat (Rone) were harvested, and the level of shed Sdc1 is demonstrated by immunoblotting (top) and quantification (bottom). (**c**) HMEC-1 cell lysate was incubated overnight with glutathione beads coated with GST or GST-S1ED and captured VLA-4 or VEGFR2 was detected by immunoblotting. (**d**) Sdc1 in HPSE^high^ or HMEC-1 cell lysates or conditioned media is analyzed for the presence of HS or CS by enzymatic treatment with bacterial HPIII or chondroitin ABC lyase (ABCase).

**Figure 8:**
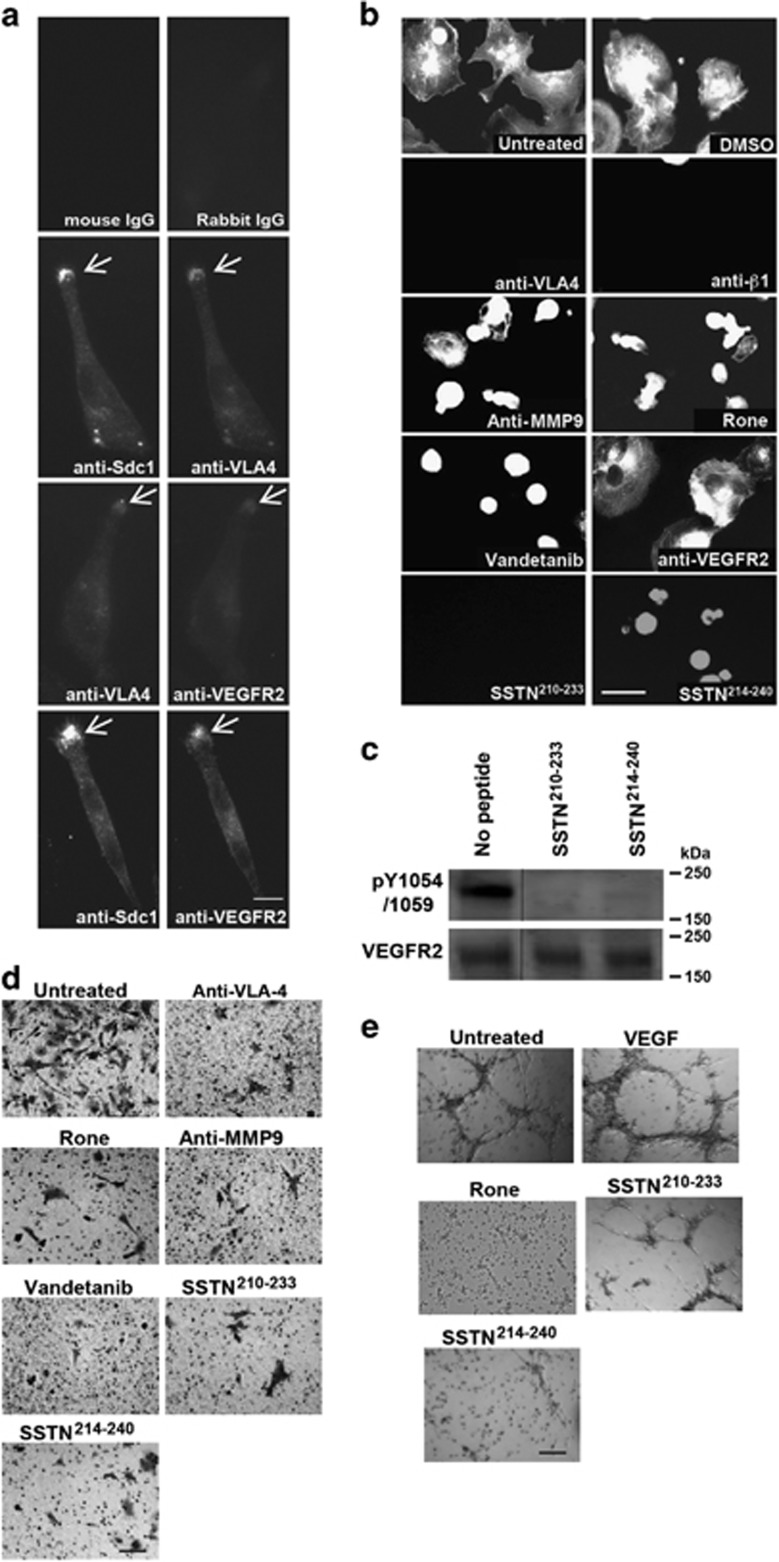
The Sdc1-coupled VEGFR2-VLA-4 complex induces angiogenesis in endothelial cells. (**a**) HMEC-1 cells were plated on the IIICS FN fragment for 5 h and double stained for VLA-4 and Sdc1, VEGFR2 and Sdc1, or VLA-4 and VEGFR2 (bar=10 μm). (**b**) HMEC-1 cells plated on IIICS were treated with DMSO vehicle, VLA-4 blocking antibody (P1H4), 10 μg/ml β1-integrin blocking antibody mAb13, MMP-9 blocking antibody, Roneparstat (Rone) (125 μg/ml), Vandetanib, VEGFR2 blocking antibody, 30 μM SSTN^210-233^ or SSTN^214-240^ (bar=50 μm). (**c**) HMEC-1 cells were plated on IIICS for 2 h in the presence of 10 μM of SSTN^210-233^, or SSTN^214-240^. Lysates were probed on immunoblots with antibodies against p1054/1059 of VEGFR2 and total VEGFR2. Note that other intervening lanes in the blot were removed. (**d**) Sixteen-hour transfilter migration assays toward GST-IIICS were performed for HMEC-1 cells in the presence of VLA-4 blocking antibody, Roneparstat (Rone), MMP-9 blocking antibody, Vandetanib, or 30 μM SSTN^210-233^ or SSTN^214-240^ (bar=100 μm). (**e**) HMEC-1 cells (2.5 x 10^4^ cells per well) were seeded onto matrigel containing GST-IIICS and cultured in media containing 20 ng/ml VEGF in the absence or presence of Roneparstat (Rone), or 30 μM of SSTN^210-233^, or SSTN^214-240^ for 24 h. Images of random fields were taken at 100x to observe capillary network formation (bar=250 μm).

VLA-4 and VEGFR2 are normally expressed on vascular endothelial cells,^46, 47, 48, 49^ suggesting that the HPSE-induced mechanism observed in myeloma may have a role in angiogenesis as well. Testing this, we find that HMEC-1 cells express levels of HPSE equivalent to the HPSE^high^ myeloma cells (Figure 7a) and that conditioned medium from the HMEC-1 cells contains shed Sdc1, which is abolished upon treatment with the HPSE inhibitor Roneparstat (Figure 7b). Furthermore, recombinant GST-S1ED captures VLA-4 and VEGFR2 from HMEC-1 cell lysates (Figure 7c), similar to capture observed in myeloma cells. A potential difficulty in extending this mechanism to endothelial cells, however, is that endothelial Sdc1, unlike myeloma, is variably decorated with chondroitin sulfate (CS) at sites that either immediately flank (ExSG^207^) or are within (ExSG^217^) the VLA-4/VEGFR2 binding region and thus may prevent receptor capture (Figure 7d). However, we find that whereas cell surface Sdc1 contains abundant CS, Sdc1 shed by HMEC-1 cells lacks CS, suggesting that a CS-free population exists that participates in this mechanism (Figure 7d). Indeed, HMEC-1 cells spread rapidly on the IIICS fragment of FN, which contains the VLA-4 binding site, and VLA-4, VEGFR2 and Sdc1 colocalize at the leading edge of the cells (Figure 8a). Binding and spreading is completely dependent on VLA-4, as inhibition of this integrin with VLA-4 or β1-integrin-specific antibody prevents adhesion (Figure 8b). Furthermore, the VLA-4-dependent spreading appears to depend on the HPSE-induced shedding of Sdc1 and activation of VEGFR2, as it is prevented by HPSE inhibitor, MMP-9 blocking antibody, and vandetanib, as well as by SSTN^210-233^ and SSTN^214-240^ (Figure 8b). HMEC-1 cells plated on IIICS activate VEGFR2, as observed by monitoring pY1054/1059, and this activation is also disrupted by SSTN^210-233^ and SSTN^214-240^ (Figure 8c). However, as observed with the CAG myeloma cells, blocking VEGF binding using VEGFR2 blocking antibody is without effect (Figure 8b). Furthermore, HMEC-1 cell invasion through transwells coated with FN is disrupted by blockade of VLA-4, inhibition of HPSE, blockade of Sdc1 shedding, blockade of VEGFR2 activation or blockade with SSTN^210-233^ or SSTN^214-240^ (Figure 8d). Finally, we tested the ability of the peptides to block angiogenesis using an *in vitro* tube formation assay (Figure 8e). HMEC-1 cells plated overnight on matrigel containing 100 μg/ml IIICS form the expected honeycomb network of endothelial tubules. Tubule formation on this matrix is not enhanced by VEGF. However, addition of the HPSE inhibitor Roneparstat, SSTN^210-233^ or SSTN^214-240^ blocks tube formation, implicating HPSE-mediated shedding of Sdc1 and its coupling of VEGFR2 to VLA-4 in angiogenesis.

## Discussion

Poor outcome in multiple myeloma is linked to high expression of HPSE and correspondingly high serum levels of shed Sdc1.^6, 10, 11, 12, 15, 21^ Here, we show using myeloma cells, and extending the finding to endothelial cells as well, that an active motif in shed Sdc1 promotes an invasive phenotype by coupling VEGFR2 to VLA-4. Coupling traces to an active site, amino acids 210–236, in the Sdc1 ectodomain that is fully functional only when the syndecan is shed. Its binding to VEGFR2 and/or VLA-4 can be mimicked by short peptides encompassing all or parts of this sequence that act as activators or inhibitors (synstatins) of this mechanism. Although typically expressed on vascular endothelial cells, VEGFR2 is aberrantly expressed in many tumors, including multiple myeloma.^50, 51, 52^ Prior reports have suggested an interaction of Sdc1 with VEGFR2 in myeloma-induced angiogenesis and vascular mimicry in melanoma;^53, 54, 55^ our work shows that this interaction is direct.

**Figure 9:**
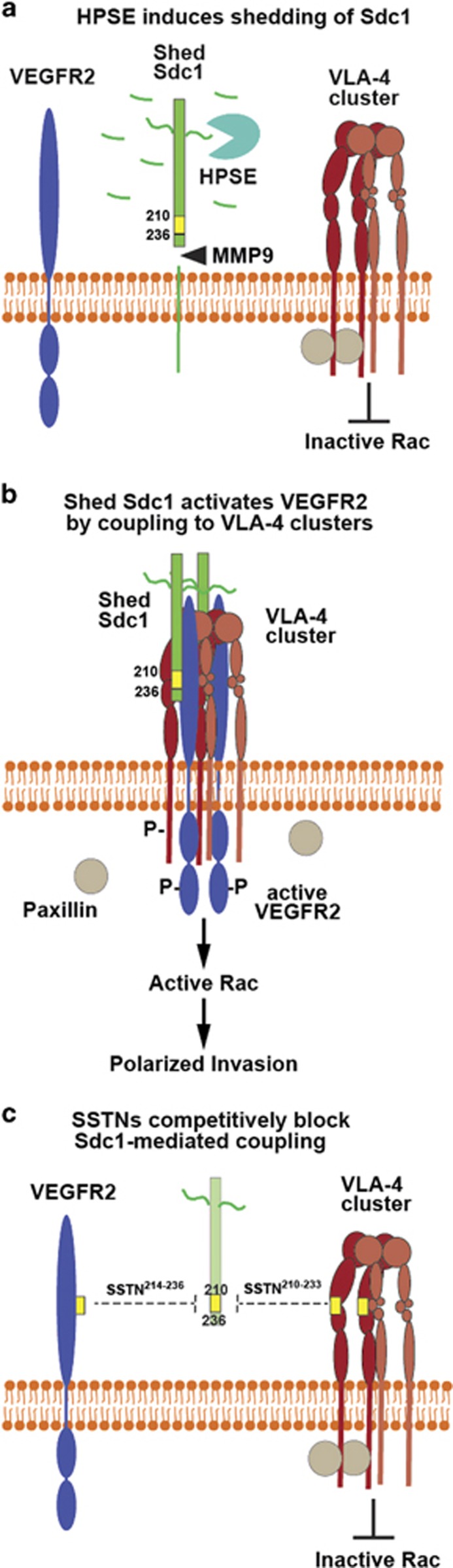
Model—postulated mechanism for polarized invasion mechanism induced by HPSE. (**a**) Clustering of VLA-4 to membrane domains leads to increased avidity and strengthening of adhesion to FN or VCAM-1. Paxillin bound to the alpha4 cytoplasmic domain causes inherent inhibition of Rac GTPase at this site. Trimming of the HS chains on Sdc1 facilitates its recognition and shedding by MMP-9. (**b**) Sdc1 freed of its membrane anchorage engages VEGFR2 and VLA-4 via its juxtamembrane receptor capture site (amino acids 210–236), coupling VEGFR2 to the VLA-4 clusters. VEGFR2 activated by the Sdc1-mediated clustering event initiates downstream signaling that displaces paxillin, allows localized activation of Rac GTPase and triggers polarized migration. (**c**) Synstatin peptides that bind either VEGFR2 alone (SSTN^210-233^) or VLA-4 alone (SSTN^214-236^) compete for Sdc1-mediated receptor capture and block the activation mechanism.

The bone marrow microenvironment is enriched in ligands for VLA-4, including VCAM-1 on endothelial cells, and VCAM-1 and FN found on stromal cells and in the matrix, respectively.^29, 30^ VLA-4 is known to cause directed cell migration in a variety of cell types, especially cells of the immune and vascular system.^48, 56, 57, 58, 59, 60, 61^ Endothelial cells and T cells subjected to shear flow re-orient, polarize and migrate in the direction of flow, localizing VLA-4 to the leading edge of the cell where it regulates activation of Rac1, actin cytoskeleton re-organization and protrusive membrane activity.^56, 57, 58, 62, 63, 64^ A key feature of this mechanism is the unique binding of paxillin to the α4 integrin cytoplasmic domain, allowing it to suppress Rac1 activation at the adhesion site.^65^ However, PKA-mediated phosphorylation of the integrin displaces paxillin and relieves its inhibition of Rac1.^58, 64^ This causes directional migration because the phosphorylation mechanism is localized to the leading edge, whereas Rac1 inhibition is maintained at lateral cell borders. A major question that remains unanswered is how PKA is activated specifically at the leading edge of the cell. We speculate that in myeloma cells and vascular endothelial cells coupling of active VEGFR2 to VLA-4 via shed Sdc1 may be one such mechanism (see model in Figure 9).

The affinity of integrins for their ligands is mediated partly by their activation and partly by avidity, a consequence of integrin clustering.^66^ Our finding that VEGFR2 is activated by Sdc1 only on adherent cells suggests that clustering of the integrin serves to cluster and activate VEGFR2. Shed Sdc1 ectodomain appears to re-localize VLA-4 from the uropod, where it is found with native Sdc1,^44, 45^ to the leading edge where it localizes with active VEGFR2. This ligand-independent mechanism may explain prior reports of VEGF-independent VEGFR2 activation, including during endothelial cell response to shear stress,^67, 68, 69, 70^ as clustering of the integrin and kinase likely leads to VEGFR2 transphosphorylation within the receptor complex. Activation of VEGFR2 by coupling it to the integrin appears critical, as VEGF alone does not suffice. This suggests that VEGFR2 kinase must act locally, either targeting a component of the integrin focal complex, or localizing additional signaling components to the adhesion site.

This is the third set of receptors, in each case a receptor tyrosine kinase and an integrin, organized by Sdc1. IGF-1R and the αvβ3 or αvβ5 integrin are captured by a distal site (amino acids 93–120 in hSdc1) in tumor cells and activated vascular endothelial cells; similar to the mechanism described herein, IGF-1R activation is independent of IGF1 and instead relies on Sdc1 clustering to sites of matrix adhesion.^26, 28, 71^ A synstatin peptide that competes for this capture site (SSTN^93-120^) disrupts angiogenesis and tumor growth *in vivo.*^26, 28^ The juxtamembrane organizer site described here (amino acids 210–236) that captures VEGFR2 and VLA-4 is part of a multifunctional site, as it overlaps with a motif (amino acids 210–240) that captures HER2 and α3β1 integrin in epithelial cells,.^25^ A multifunctional juxtamembrane site exists in Sdc4 as well^72, 73^ that mediates β1-integrin-dependent attachment of fibroblasts^74, 75, 76^ and captures and activates EGFR and α3β1 integrin in epithelial cells.^25^ A SSTN representing this site (SSTN^87-131^) blocks the motility of EGF-stimulated epithelial cells.^25^

Peptides that bind only the α4β1 integrin (SSTN^210-233^) or VEGFR2 (SSTN^214-240^) act as competitive inhibitors of myeloma or endothelial cell invasion with possible roles as cancer therapeutics. An unanticipated finding is that SSTN^210-233^ appears to not only disrupt coupling of VEGFR2 to α4β1 integrin by shed Sdc1, but also disrupts VLA-4-mediated adhesion that relies on membrane-bound Sdc1 and is independent of HPSE. This suggests an additional role for Sdc1, to be investigated further, in integrin activation or avidity. This HPSE- and VEGFR2-independent mechanism is likely active in other cells that express VLA-4, especially cells in the immune system. SSTN^210-233^ may also have additional antitumor activities in myeloma, including blocking cell adhesion-mediated drug resistance (CAM-DR)^77, 78, 79, 80^ and blocking the activation of osteoclasts and their insidious bone erosion that characterizes this disease.^81, 82^ A number of other VLA-4 specific inhibitors (for example, Natalizumab) are currently in clinical development to target these mechanisms (reviewed in Shishido *et al.*^83^).

SSTN^214-240^, which targets the ability of the syndecan to capture VEGFR2, appears to be highly specific for invasion because of its ability to block directional cell migration. This requires release of the syndecan from its membrane anchorage, perhaps re-orienting to fit a binding pocket on VEGFR2, or freeing it to translocate to another site, for example, from the lagging to the leading edge of the cell. This is a tightly regulated process that likely governs the transition from stationary to invasive behavior. Decoration of Sdc1 with HS and, as our current work now suggests, with CS, regulates the process by preventing the shedding. Sdc1 has long been known to contain both HS and CS chains,^84^ but a role for the CS has not been described previously. Our findings suggest that its attachment to the core protein in or near the VEGFR2/VLA-4 engagement site may prevent shedding, and may also prevent capture of these receptors. Whether CS degradation by cell surface enzymes can serve to regulate this mechanism, or whether it depends on regulated chain attachment during synthesis, is completely unknown. It is noteworthy that myeloma cells, which are likely to be highly dependent on CS-free Sdc1, express only minimal CS on the syndecan, if any.^19^

Another critical feature of the invasion program is upregulation of HPSE expression along with MMP-9 and other matrix metalloproteinases that cleave the syndecan, and erode the matrix.^10, 17^ Our findings provide new mechanistic insight into how HPSE, which is upregulated in essentially all major forms of cancer,^5, 85, 86, 87^ wields its powerful impact on tumor progression and provides further impetus for development of HPSE inhibitors such as Roneparstat (formerly named SST0001), which has shown preclinical efficacy against myeloma, pediatric sarcoma and pancreatic carcinoma and is now being tested in phase I/II clinical trials in myeloma patients.^35, 36, 37, 88, 89^ Roneparstat is a glycol split-modified heparin, a close mimic of HS, which lacks the potent anti-coagulant activity of heparin. Other HPSE inhibitors based on sulfated polysaccharides include PI-88, M402, maltohexose sulfate and PG545, which also display anti-metastatic, anti-angiogenic and anti-inflammatory activities and are being investigated in clinical trials.^6, 90, 91, 92, 93^

In summary, we have identified a mechanism by which HPSE expression in myeloma and endothelial cells leads to activation of VEGFR2 and an invasive phenotype. SSTN peptides that engage VLA-4 or VEGFR2 act as potent inhibitors of this mechanism and have significant potential, along with inhibitors of HPSE enzyme activity, as therapeutics to target cancer and other diseases that depend on this mechanism.

## Materials and methods

### Reagents

HPSE inhibitor Roneparstat^35, 36^ (formerly known as SST0001) was provided by Sigma-Tau Research Switzerland S.A. (Mendrisio, Switzerland). OGT 2115 was from TOCRIS Bioscience (Minneapolis, MN, USA). Our use of recombinant HPSE was described previously.^17^ Peptides were from LifeTein (Plainfield, NJ, USA), Vandetanib (ZD6474) from LC Laboratories (Woburn, MA, USA) and VEGF165 from PeproTech (Rocky Hill, NJ, USA). Recombinant GST-mouse-S1ED was prepared as described.^94, 95^ Anti-GM130 (cat. 610822) was from BD Biosciences (San Diego, CA, USA). Anti-VEGFR2 (cat. 2479S) was from Cell Signaling Technology (Danvers, MA, USA), anti-VEGFR2 (cat. 05–554) and anti-α4 integrin (cat. MAB16983Z) were from Millipore (Billerica, MA, USA), anti-mouse α4 integrin (cat. 553313 was from BD Biosciences (San Jose, CA, USA)), anti-β1 integrin (clone mAb13) was kindly provided by Dr Steve Akiyama (NIEHS), polyclonal antibodies against human Sdc1 (refs^94^, ^96^) and monoclonal anti-mouse Sdc1 281.2 (ref. 97) were described previously. Secondary antibodies were from Jackson ImmunoResearch (West Grove, PA, USA). Rabbit polyclonal anti-α4 integrin and anti-pY^1054/1059^ VEGFR2 (cat. 441047G) were from Fisher (Grand Island, NY, USA). Anti-MMP-9 (cat. IM09T) inhibitory antibody was from Calbiochem (La Jolla, CA, USA). Matrigel was from Corning Incorporated Life Sciences (Tewksbury, MA, USA). His-recombinant VEGFR2 protein was from ACRO Biosystems (Newark, DE, USA).

### Molecular biology

The expression of Sdc1 in pcDNA3 vector was described previously.^26, 28^ Small interfering RNA (nucleotide annotation: 5'-GGAGGAATTCTATGCCTGA-3') specific for human Sdc1 was designed by Ambion (Austin, TX, USA). For transfection, 60 pmol small interfering RNA or 5 ug of DNA was added to 10^6^ cells in six-well plates using Lipofectamine 2000 and Opti-MEM transfection medium (Invitrogen, Carlsbad, CA, USA) lacking serum and antibiotics followed by 3 ml complete growth medium after 5 h.

### Cell adhesion and spreading assays

The derivation and culture of CAG HPSE^low^ and HPSE^high^ myeloma and HMEC-1 cells are described previously.^71, 98^ P3-X63-Ag8 cells obtained from ATCC (Rockville, MD, USA) were grown in RPMI supplemented with 10% fetal bovine serum at 37 °C and 92.5% air/7.5% CO_2_. All cells are routinely screened for mycoplasma contamination. Nitrocellulose-treated slides^99^ were coated for 2 h at 37 °C with 40 μg/ml FN (kindly provided by Dr Donna Peters, University of Wisconsin-Madison) or FN IIICS fragment,^100^ or 5 μg/ml recombinant VCAM-1/Fc chimera (R&D Systems, Minneapolis, MN, USA) in calcium and magnesium-free phosphate-buffered saline (PBS) (CMF-PBS; 135 mM NaCl, 2.7 mM KCl, 10.2 mM Na_2_HPO_4_-7H_2_O and 1.75 mM KH_2_PO_4_, pH 7.4), then blocked with RPMI 1640 containing 1% heat-denatured bovine serum albumin (plating medium). Cells in plating medium were allowed to spread for 2.5 h at 37 °C, fixed in 4% paraformaldehyde (Electron Microscopy Sciences, Hatfield, PA, USA) in CMF-PBS, labeled with rhodamine phalloidin (Invitrogen) and imaged with a Nikon Microphot FX microscope using a 20X objective (Ex 541–551, DM 580, Barrier 590 (Nikon, Melville, NY, USA)), Photometric CoolSnap ES camera (Photometics, Tuscon, AZ, USA), and version 7.7.3.0 Metamorph Imaging software (Molecular Devices, Downington, PA, USA). All images represent results from triplicate wells and three independent experiments. Note that experiments are routinely conducted on both FN and VCAM-1, with identical results. For immunostaining, fixed cells were quenched with 0.1 M glycine, permeabilized (in select experiments) with CMF-PBS containing 0.1% Triton X-100 for 3 min at room temperature (RT) and blocked with 5% bovine serum albumin in CMF-PBS. Staining was for 2 h at RT, followed by rinses in CMF-PBS, and incubation for 1 h with 1:100 Alexa-488-conjugated goat anti-mouse IgG (H+L) F(ab')_2_ or Alexa-546-conjugated goat anti-rabbit IgG (H+L) F(ab')_2_ (Molecular Probes, Waltham, MA, USA) (1:100 dilution) in blocking buffer. The nucleus was stained with DAPI (1:1000 dilution) in CMF-PBS at RT for 10 min. Images were processed using Adobe Photoshop (Adobe Systems, San Jose, CA, USA).

### Migration assays

The bottom chambers of Transwell filter chambers (8 μm pores; Corning, Tewksbury, MA, USA) were coated with 40 μg/ml of FN or 100 μg/ml of GST-IIICS (prepared as described).^100^ Cells (5 × 10^5^) were placed in the upper chamber and incubated for 16 h at 37 °C. Cells on the bottom of the filter were fixed and stained with 0.1% crystal violet, and five random fields were imaged and counted. For live imaging, cells were plated in FN-coated 48-well plates in plating medium and allowed to attach and spread for 2.5 h. Attached cells were observed at 37 ^o^C on a Nikon Eclipse TE2000U equipped with environmental chamber using a PlanApo 20X objective (0.75 numerical aperture) and a Photometrics CoolSnap ES camera. Cells were tracked with Metamorph and images were collected at 10-min intervals over 3 h.

### Immunoprecipitation and GST-S1ED pull-down assay

HPSE^low^ cells were plated on FN in the presence of 4 μg/ml GST or GST-S1ED for 2.5 h, were washed with CMF-PBS, then lysed in ice-cold buffer (0.5% Triton X-100, 50 mM HEPES, 50 mM NaCl and 10 mM EDTA (pH 7.4)) containing 1:1000 protease inhibitor mixture III (Calbiochem). Pre-cleared lysates were incubated at 4 °C overnight with either anti-α4 integrin (mAb 44H6) or VEGFR2 (mAb 55B11) or nonspecific mouse IgG, precipitated on protein G beads, and analyzed on western blots as described.^30^ Bands were detected with AP-conjugated antibody and ECF reagent (Amersham Pharmacia, Pittsburgh, PA, USA) on a Typhoon Trio Variable Mode Imager (GE Healthcare, Pittsburgh, PA, USA).

For GST-S1ED pull-down, lysates were incubated at 4 °C overnight with either 3 μM GST or 3 μM GST-S1ED in the presence of 30 μM S1ED peptides. GST or GST-S1ED was captured by glutathione sepharose, washed with ice-cold lysis buffer and CMF-PBS before western blot analysis.

To isolate shed Sdc1, HPSE^high^, HPSE^high^ transfected with mSdc1 or HMEC-1 cells were grown for 72 h, then conditioned media and cell lysates were collected, and Sdc1 captured on protein G beads with anti-human or mouse Sdc1 at 4 °C overnight. Decoration with HS or CS was analyzed by treatment with 0.2 U/ml of HPIII or chondroitin ABC lyase followed by size shift analysis on western blots. Or, capture of VLA-4/VEGFR2 by the shed Sdc1 was analyzed by re-incubating the beads with HPSE^low^ cell lysates at 4 °C overnight and analysis on western blots.

### Tube formation assay

HMEC-1 cells (2.5 × 0^4^ cells per well) were seeded onto matrigel containing 100 μg/ml GST-IIICS and cultured in serum-free MCDB131 medium containing VEGF in the absence or presence of 125 μg/ml Roneparstat or 30 μM of SSTN peptides for 24 h. Pictures were taken of random fields.

### Statistical analysis

Experiments were repeated a minimum of three times. Comparisons between treated and untreated groups displayed uniform variance and were compared using the unpaired one-tail *t*-test. *P*-values of <0.05 were considered significant. All *in vitro* data are represented as mean±s.d.
