# Supplementary figures and images for: Heparanase-induced shedding of syndecan-1/CD138 in myeloma and endothelial cells activates VEGFR2 and an invasive phenotype: prevention by novel synstatins
Source: Oncogenesis. 2016 Feb 29;5(2):e202–. doi: 10.1038/oncsis.2016.5 (PMC5154350; doi:10.1038/oncsis.2016.5)

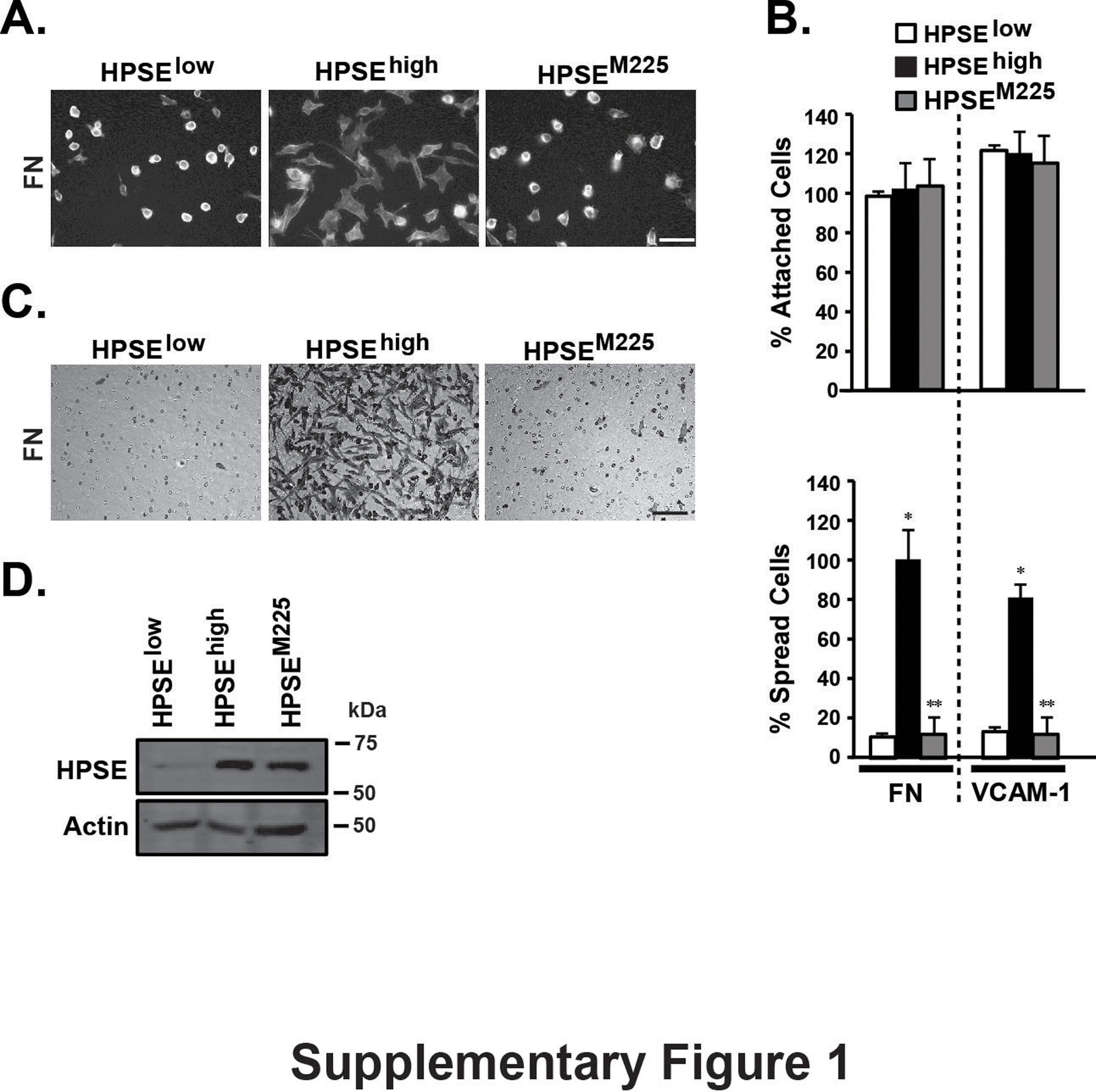

Supplement: Supplementary Figure 1 [file oncsis20165x1.tif]

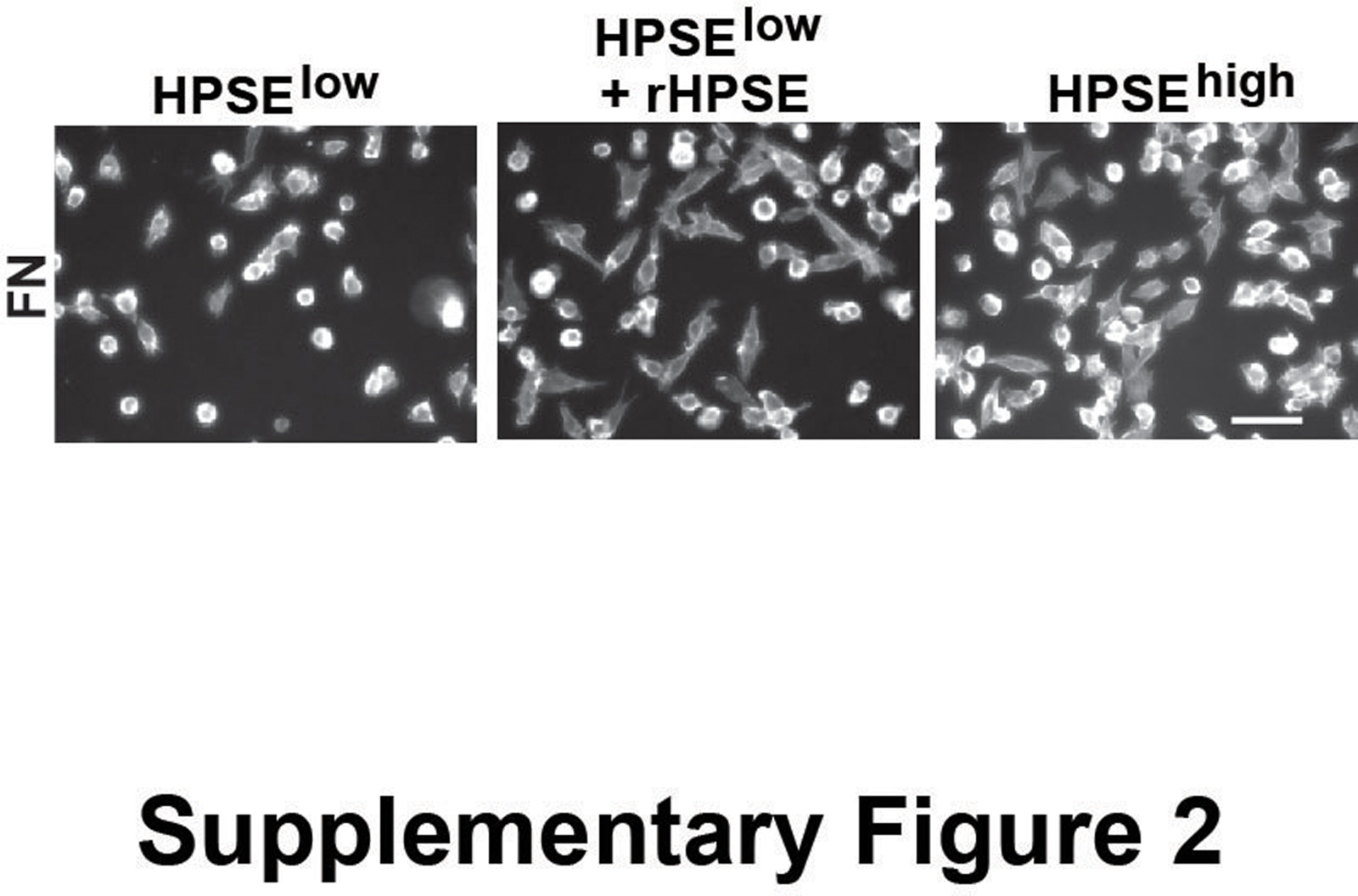

Supplement: Supplementary Figure 2 [file oncsis20165x2.tif]

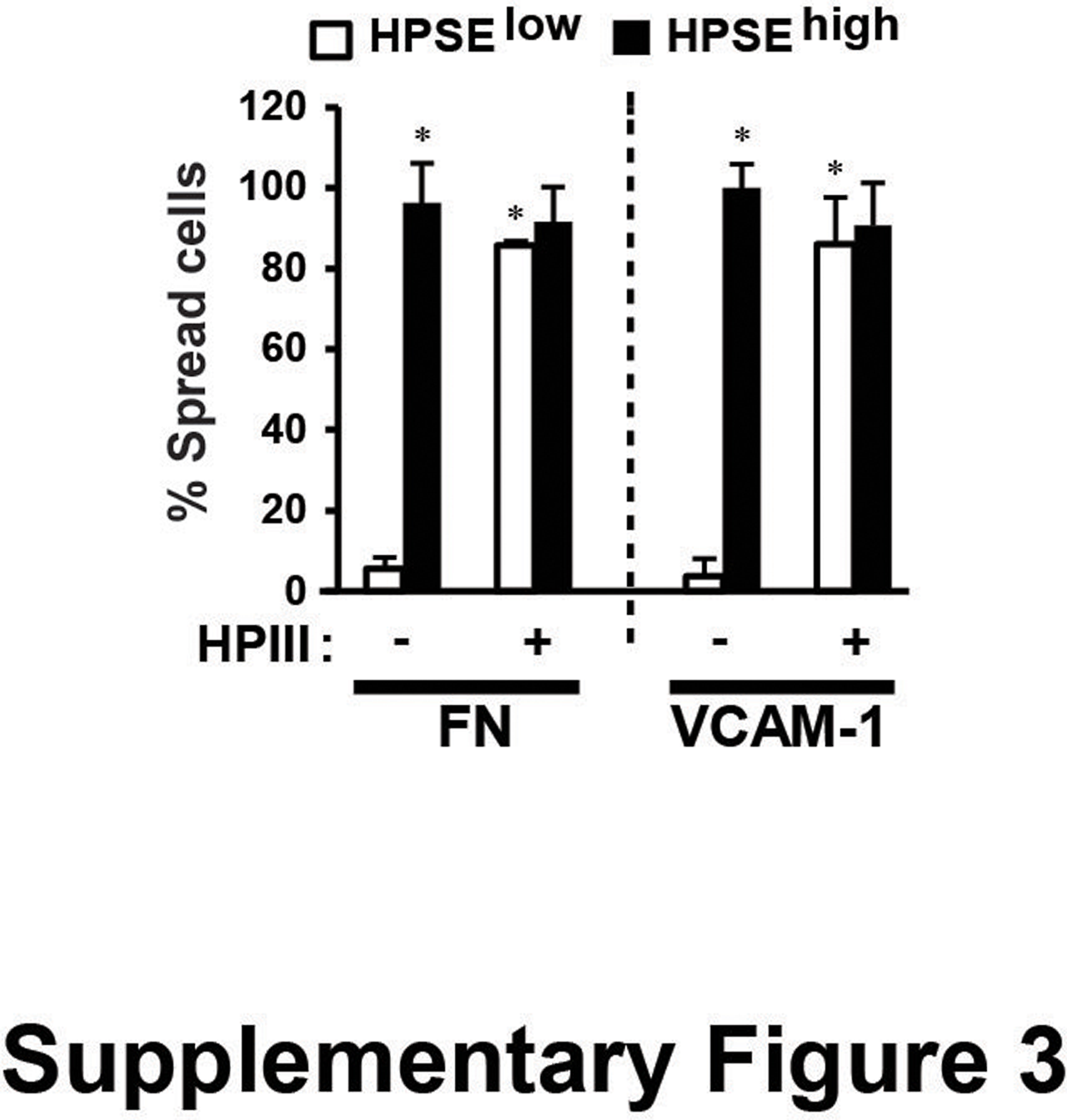

Supplement: Supplementary Figure 3 [file oncsis20165x3.tif]

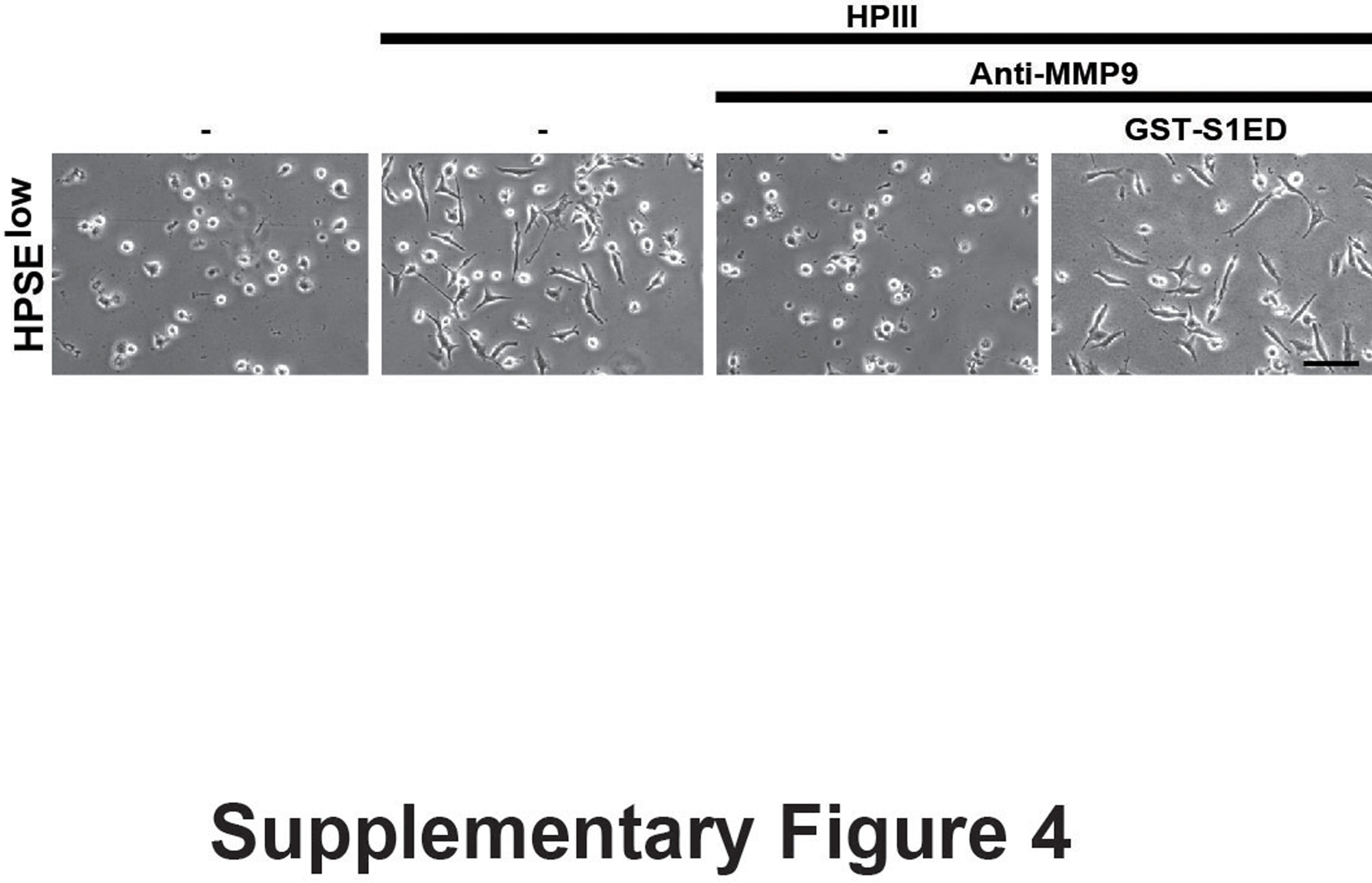

Supplement: Supplementary Figure 4 [file oncsis20165x4.tif]

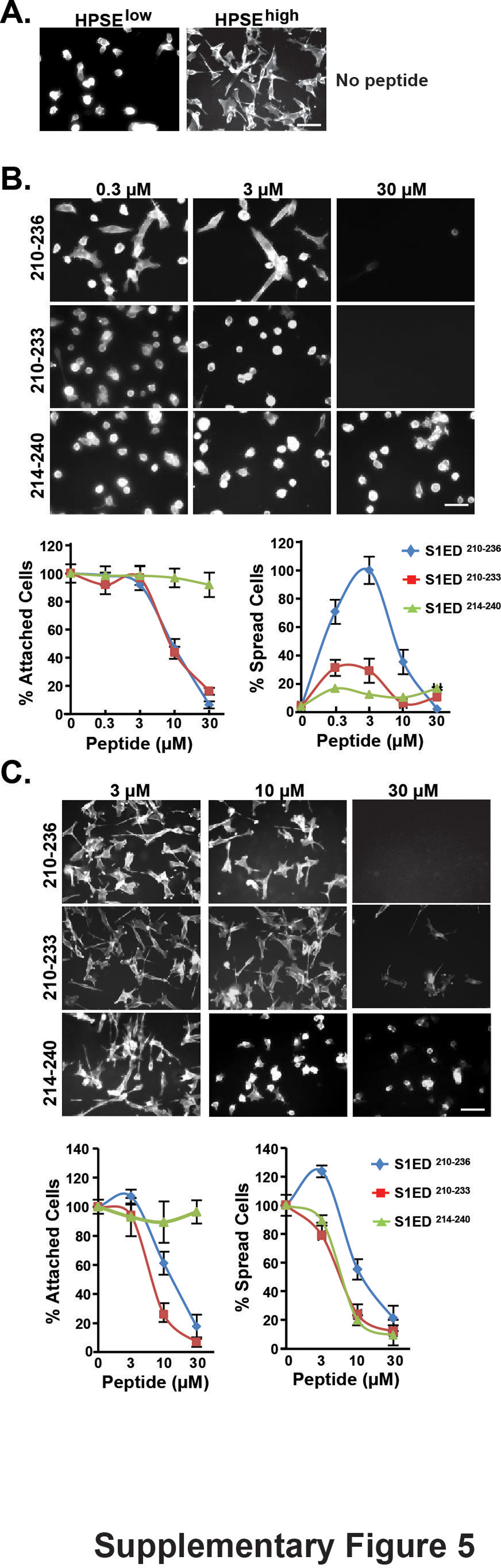

Supplement: Supplementary Figure 5 [file oncsis20165x5.tif]

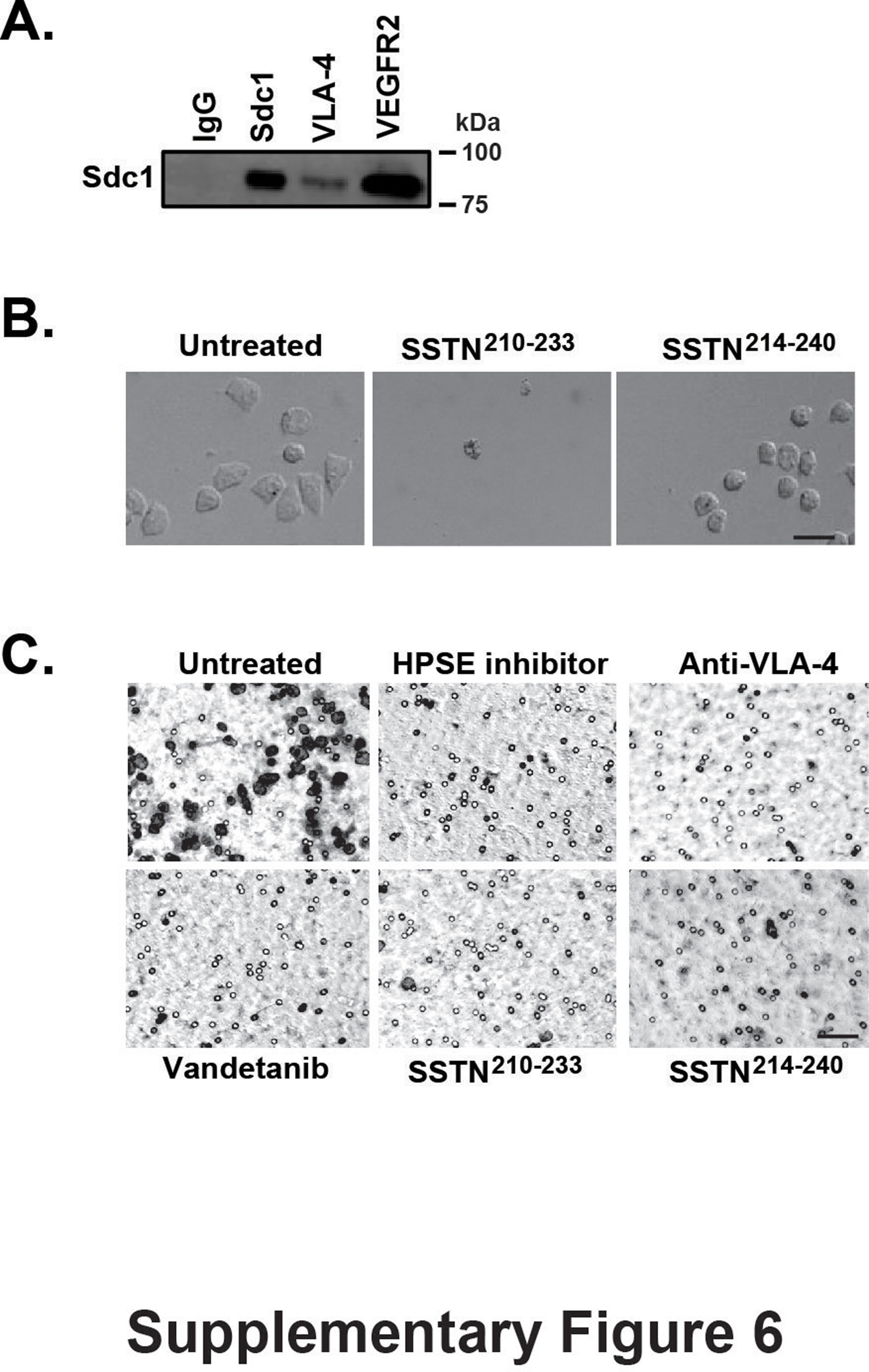

Supplement: Supplementary Figure 6 [file oncsis20165x6.tif]
