# Supplementary material for: Heparanase-induced shedding of syndecan-1/CD138 in myeloma and endothelial cells activates VEGFR2 and an invasive phenotype: prevention by novel synstatins
Source: Oncogenesis. 2016 Feb 29;5(2):e202–. doi: 10.1038/oncsis.2016.5 (PMC5154350; doi:10.1038/oncsis.2016.5)
Supplement: Supplementary Figure Legends [file oncsis20165x7.doc]

**Supplementary Figures:**

**S1.** **The HPSE-induced polarized invasive phenotype is mediated by HPSE enzymatic effect. (A)** HPSE^low^, HPSE^high^ , or HPSE^M225^ cells were plated on FN and stained with fluorescent phalloidin to visualize the cells (Bar = 50 μm); (B) Quantification of HPSE^low^, HPSE^high^, or HPSE^M225^ cell attachment or spreading on FN or VCAM-1. * *P*<0.05 against HPSE^low^ cells. ** *P*<0.05 against HPSE^high^ cells; (C) of HPSE^low^, HPSE^high^, or HPSE^M225^ cell migration towards FN after 16 h migration. Cells on the bottom of the filter in five random images for each experiment are quantified as a percent of HPSE^high^ cell migration (Bar: 100 µm); (D) Lysates from HPSE^low^, HPSE^high^, or HPSE^M225^ cells were probed by immunoblotting for expression of HPSE. Actin is shown as a loading control; Error bars represent S.E.

**S2.** R**ecombinant HPSE treatment causes polarized cell spreading of CAG cells.** HPSE^high^ or HPSE^low^ cells treated with or without recombinant HPSE were plated on FN and stained with fluorescent phalloidin to visualize the cells (Bar = 50 μm).

**S3.** **Trimming HS chains by HPIII causes polarized cell spreading of CAG cells. H**PSE^low^ or HPSE^high^ cells pretreated with HPIII for 2.5h were plated on FN or VCAM-1 and cell spreading was quantified and expressed as a percent of HPSE^high^ cell spreading on VCAM-1. * *P*<0.05 against HPSE^low^ cells; Error bars represent S.E;

**S4.** **HPSE^low^ cell spreading induced by HPIII is inhibited by MMP-9 inhibitor, and rescued by GST-S1ED. H**PSE^low^ cells pretreated with HPIII for 2.5h were plated on VCAM-1 in the absence or presence of MMP9 blocking antibody with or without GST-S1ED (Bar=100 µm).

**S5.** **SSTN peptides specific for VEGFR2 or VLA-4 inhibit the HPSE-induced invasive phenotype.** (A) Images of **H**PSE^low^ or HPSE^high^ cells plated on FN for 2.5 h; (B) **H**PSE^low^ cells were plated on FN and treated with 0, 0.3, 3, 10 or 30 μM of S1ED^210-236^, S1ED^210-233^, or S1ED^214-240^, then fixed and stained with fluorescent phalloidin. Cells from five random images were quantified for cell attachment and cell spreading compared to the control cells shown in (A) (Bar =50 μm); (C) HPSE^high^ cells were plated and treated as in (B) (Bar = 50 μm). Note that spreading is relative to untreated HPSE^high^ cells, which is set to 100%. Thus, enhanced spreading due to peptide addition can result in greater than 100% spreading. Error bars represent S.E.

**S6.** **SSTN peptides inhibit P3-X63-AG8 myeloma cell attachment, spreading and invasion.** (A) P3-X63-AG8 myeloma cells plated on FN, then whole cell lysates were subjected to immunoprecipitation with anti-Sdc1, anti-VEGFR2, anti-VLA-4, or nonspecific IgG. Precipitated Sdc1 was detected by immunoblotting with anti-Sdc1 antibody (282.1); (B) P3-X63-AG8 cells were plated on VCAM-1 with or without 30 μM of S1ED210-233, or S1ED214-240 (Bar = 50 μm); (C) P3-X63-AG8 cells plated on VCAM-1 were treated with or without, 10 μg/ml VLA4 blocking antibody (9C10), 10 μM HPSE inhibitor OGT-2115, 10 μM Vandetanib, 30 μM S1ED^210-233^ or 30 μM S1ED^214-240^ (Bar = 100 μm).
